# Vascular plants of Reserva Biológica do Tinguá, Rio de Janeiro, Brazil: leveraging herbarium databases to address knowledge gaps in the Atlantic Forest

**DOI:** 10.3897/BDJ.13.e157961

**Published:** 2025-07-03

**Authors:** Thuane Bochorny, Alexandre Quinet, Ana Carolina D. Castello, Anderson Alves-Araújo, Andrea F. Costa, Ariane L. Peixoto, Cassia M. Sakuragui, Claudio N. Fraga, Claudenice H. Dalastra, Danilo A. Zavatin, Diego R. Gonzaga, Eliana Ramos, Eliane L. Jacques, Elsie F. Guimaraes, Elton J. Lírio, Gabriel M. Marcusso, Genise V. Sommer, George A. Queiroz, Haroldo C. Lima, Igor H. F. Azevedo, José Fernando Baumgratz, Lana S Sylvestre, Lara S. J. Deccache, Leandro L. Giacomin, Leandro C. Pederneiras, Lucas C. Marinho, Marcelo Souza, Marcus N.C. Nadruz, Marli P. Morim, Massimo G. Bovini, Miriam Kaehler, Natalia Barros, Otávio L. Marques, Pedro L. Viana, Ronaldo Vinícius-Silva, Sebastião J.S. Neto, Tatiana T. Carrijo, Rafaela C. Forzza

**Affiliations:** 1 Jardim Botânico do Rio de Janeiro, Rio de Janeiro, Brazil Jardim Botânico do Rio de Janeiro Rio de Janeiro Brazil; 2 Universidade do Estado de Minas Gerais, Ituiutaba, Brazil Universidade do Estado de Minas Gerais Ituiutaba Brazil; 3 Universidade Federal da Bahia, Salvador, Brazil Universidade Federal da Bahia Salvador Brazil; 4 Universidade Federal do Rio de Janeiro, Rio de Janeiro, Brazil Universidade Federal do Rio de Janeiro Rio de Janeiro Brazil; 5 Universidade Federal do Rio Grande do Sul, Porto Alegre, Brazil Universidade Federal do Rio Grande do Sul Porto Alegre Brazil; 6 Universidade de São Paulo, São Paulo, Brazil Universidade de São Paulo São Paulo Brazil; 7 Universidade Federal do Oeste do Pará, Santarém, Brazil Universidade Federal do Oeste do Pará Santarém Brazil; 8 Instituto Nacional da Mata Atlântica, Santa Teresa, Brazil Instituto Nacional da Mata Atlântica Santa Teresa Brazil; 9 Universidade Federal Rural do Rio de Janeiro, Seropédica, Brazil Universidade Federal Rural do Rio de Janeiro Seropédica Brazil; 10 Universidade do Estado do Rio de Janeiro, Rio de Janeiro, Brazil Universidade do Estado do Rio de Janeiro Rio de Janeiro Brazil; 11 Universidade Federal de Mato Grosso, Cuiabá, Brazil Universidade Federal de Mato Grosso Cuiabá Brazil; 12 Universidade Federal da Paraíba, João Pessoa, Brazil Universidade Federal da Paraíba João Pessoa Brazil; 13 Universidade Federal Fluminense, Campos dos Goytacazes, Brazil Universidade Federal Fluminense Campos dos Goytacazes Brazil; 14 Universidade Federal do Maranhão, São Luís, Brazil Universidade Federal do Maranhão São Luís Brazil; 15 Universidade Federal do Paraná, Curitiba, Brazil Universidade Federal do Paraná Curitiba Brazil; 16 Agroflor Engenharia e Meio Ambiente, Viçosa, Brazil Agroflor Engenharia e Meio Ambiente Viçosa Brazil; 17 Universidade Federal do Espírito Santo, Alegre, Brazil Universidade Federal do Espírito Santo Alegre Brazil; 18 Instituto Chico Mendes de Conservação da Biodiversidade, Prado, Brazil Instituto Chico Mendes de Conservação da Biodiversidade Prado Brazil

**Keywords:** Atlantic Forest, Catálogo de Plantas das Unidades de Conservação do Brasil, Conservation, Protected Areas, Taxonomy Inventories, Threatened Species

## Abstract

**Background:**

The Reserva Biológica do Tinguá is a protected area located in Rio de Janeiro state, Brazil. It is part of the Atlantic Forest domain and primarily features Dense Ombrophilous Forest, ranging from lowland to submontane, montane, and highland vegetation types. The Reserva Biológica do Tinguá is critically important for conservation, ranking among the priority areas for protecting the biodiversity of the Atlantic Forest, as well as local water supply. Understanding and accessing the floristic list within the regions is essential to developing effective conservation strategies. We utilize herbaria databases to create a comprehensive list of plant species based on revised taxonomic data. The updated list of vascular plants recorded in Rebio Tinguá is available in the “Catálogo de Plantas das Unidades de Conservação do Brasil” and is presented here with additional details on species richness, endemism, and conservation status.

**New information:**

The Reserva Biológica do Tinguá contains 1,301 species of vascular plants, including 1,133 angiosperms, one gymnosperm, and 167 are ferns and lycophytes. Of these species, 52,2% are endemic to the Atlantic Forest. There are 97 threatened species, of which five are considered Critically Endangered (CR), 57 Endangered (EN), and 36 Vulnerable (VU) at national level. Among the threatened species, 86 are endemic to the Atlantic Forest. The number of records and species richness in this area are notably high, comparing to Atlantic Forest standards. Protecting areas like the Reserva Biológica do Tinguá in densely populated urban centers presents considerable challenges due to environmental degradation, including air and water pollution and extraction of natural resources. Recognizing the ecological significance and promoting floristic studies of the remaining fragments of the Atlantic Forest is essential for biodiversity conservation ensuring overall environmental integrity.

## Introduction

Habitat loss is one of the biggest threats to biodiversity worldwide (WWF 2024). Since European colonizers first arrived on the Brazilian coast 500 years ago, the Atlantic Forest was the initial area explored and where the earliest settlements were established (Dean 1996, Ribeiro et al. 2009). Historically, Brazil’s largest urban concentrations have been located within the Atlantic Forest, including major cities like Rio de Janeiro, Salvador, and São Paulo (Rezende et al. 2018). While 90% of the Atlantic Forest’s population resides in urban centers, more than half of the national land designated to horticulture is also located within this domain (Pinto et al. 2012). The urbanization, industrialization, and agricultural expansion led to a loss of natural habitats and further reduced the extent of the Atlantic Forest (Joly et al. 2014, Gonçalves-Souza et al. 2025).

Despite housing 16,763 vascular plant species (including lycophytes, ferns, gymnosperms, and angiosperms), with a high level of endemism, the Atlantic Forest has continued to face increasing destruction over the past few decades (Tabarelli et al. 2012, Rezende et al. 2018), even in protected areas, where usually the main patches of mature forest remain, leading to a reduction in biodiversity and its associated ecosystem services (Amaral et al. 2025). Today, what remains of the Atlantic Forest is confined to a few Brazilian protected areas, particularly along the slopes of the Serra do Mar, Serra Geral, and Serra da Mantiqueira in the South and Southeast regions (Ribeiro et al. 2009, Tabarelli et al. 2010, Rezende et al. 2018). Additionally, isolated patches persist in the highlands and the northeastern region of Brazil, remaining in what were once biodiversity-rich areas (Joly et al. 2014, SOS Mata Atlântica 2019). Indeed, the remaining forest fragments in the Atlantic Forest are often too small to support the long-term survival of many species (Joly et al. 2014). Consequently, it is not surprising that 2,845 plant species in the Atlantic Forest are threatened with extinction, representing 24% of all threatened species at the national level (CNCFlora 2025).

The Reserva Biológica do Tinguá (hereafter Rebio Tinguá) is a protected area in the southwest of Rio de Janeiro state, Brazil. Its vegetation consists of Dense Ombrophilous Forest, ranging from lowland to submontane, montane, and highland forest types (IBGE 2012). Due to its proximity to a large urban center, the area faces significant threats, including wood extraction and illegal hunting (MMA–IBAMA 2006). In addition, Rebio Tinguá is crossed by roads in some areas, has water collection points for distribution that have been in operation since 1877, and contains oil pipelines within its territory (Deccache et al. 2024a, MMA–IBAMA 2006). As a result, the forest vegetation is considerably altered by the proximity of these urban centers, leading to the formation of fragments, possibly increasing the edge effects on plant species in the region.

Despite the impacts suffered by Rebio Tinguá, botanical expeditions have been conducted in the area since the 19^th^ century. After the official establishment of the protected area, carried out between 1991 and 2009, various naturalists visited the region as part of projects such as the '*Projeto Paisagem e Flora da Reserva Biológica do Tinguá*' (Landscape and Flora of the Rebio Tinguá) and the '*Projeto Mata Atlântica*' (Atlantic Forest Project) (Deccache et al. 2024a).

Today, Rebio Tinguá remains one of the few forested areas in the Baixada Fluminense region, playing a crucial role in the conservation of the Atlantic Forest and as a main water supply source for the surrounding municipalities. Furthermore, since 2012, Rebio Tinguá has been part of the '*Programa de Pesquisa em Biodiversidade*' - PPBio (Biodiversity Research Program) with permanent sampling plots that were the focus of a notable recent checklist of Atlantic Forest trees in Rebio Tinguá (Iguatemy et al. 2017). This effort in this region also led to other floristic and phytosociological studies focusing on tree species (Braz et al. 2004, Sobrinho et al. 2010, Negreiros et al. 2023, Deccache et al. 2024a). In this study, we present and discuss information on the richness, endemism, and conservation status of vascular flora recorded in Rebio Tinguá.

## Sampling methods

### Step description

**Species list**

**Figure 1.**
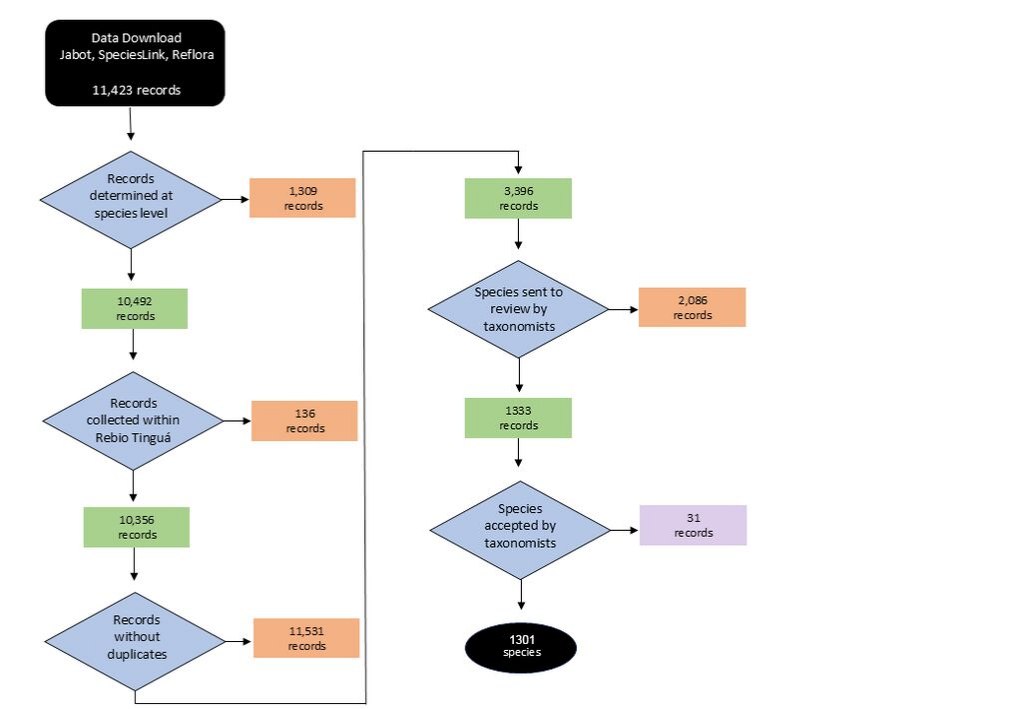
Workflow for data cleaning and elaboration of the species list for Reserva Biológica do Tinguá (Rebio Tinguá), Rio de Janeiro, Brazil. Specimens retained in the list are shown in green, while those removed are shown in orange. Specimens excluded by taxonomists are shown in purple.

The list of plant species collected in Rebio Tinguá was based on data obtained from three main databases of Brazil: Jabot Geral (Jardim Botânico do Rio de Janeiro, http://jabot.jbrj.gov.br/v3/consulta.php), Reflora (Herbário Virtual Reflora, http://reflora.jbrj.gov.br), and speciesLink (INCT Herbário Virtual da Flora e dos Fungos, http://inct.splink.org.br). The databases were accessed on October 10, 2022, and the records were filtered using the following criteria = 'Reserva Biológica do Tinguá' and 'Rebio Tinguá'. Our searches returned a total of 11,423 specimens (Jabot Geral = 5,702; Reflora = 3,322; speciesLink = 2,399; Fig. 1). We manually selected all specimens identified at specific level, which led to: Jabot Geral determined = 5,497, undetermined = 583; Reflora determined = 2,939, undetermined = 383; and speciesLink determined = 2,056, undetermined = 343; Fig. 1). We then removed duplicates based on collector name, collector number, and year of collection, and selected one record per species, prioritizing those records with digitized specimens. We also excluded records whose recorded locations were outside the boundaries of Rebio Tinguá (Fig. 1). Finally, we updated the species names according to Flora e Funga of Brasil (http://floradobrasil.jbrj.gov.br). After these corrections, we sent the preliminary list, comprising 1,333 species, to taxonomists (authors of this paper) to check and validate determinations using images in online databases. Intraspecific taxonomic categories and hybrids were not considered. The final checklist of the vascular plants from Rebio Tinguá was published by Bochorny et al. (2022) and is available in the “Catálogo de Plantas das Unidades de Conservação do Brasil” (https://catalogo-ucs-brasil.jbrj.gov.br/descr_areas.php?area=RebioTingua).

**Origin, endemism and conservation status**

We verified the origin of species (native, cultivated or non-native) and endemism to the Atlantic Forest following the Flora e Funga of Brazil website (http://floradobrasil.jbrj.gov.br). Conservation status of the species was automatically assigned using the CNCFlora (2025) public database (Official National Red List published by MMA Ordinance No. 148/2022), which serves as the IUCN SSC Brazil Plant Red List Authority (IUCN SSC BP-RLA).

## Geographic coverage

### Description

**Figure 2.**
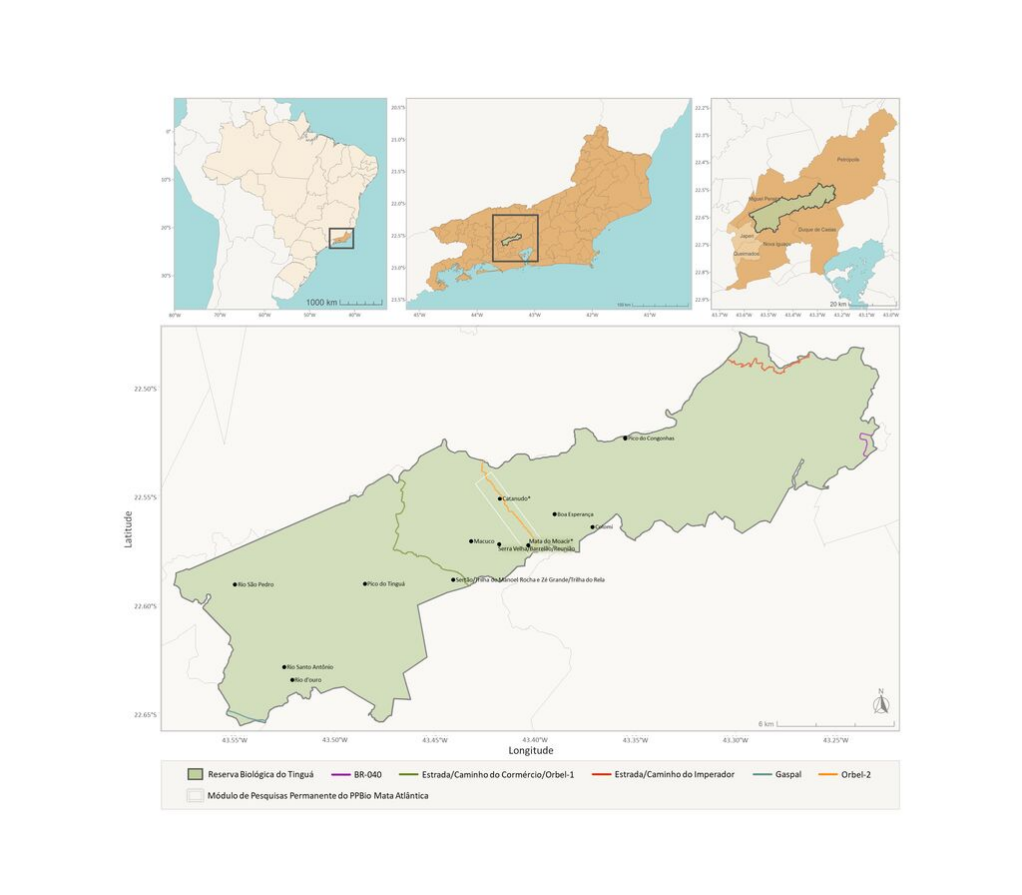
Map showing the location of the Reserva Biológica do Tinguá (Rebio Tinguá), Rio de Janeiro, Brazil, including roads within the reserve, main attractions, and PPBio (Programa de Pesquisa em Biodiversidade – Biodiversity Research Program) plots. (Map from Deccache 2023).

**Figure 3.**
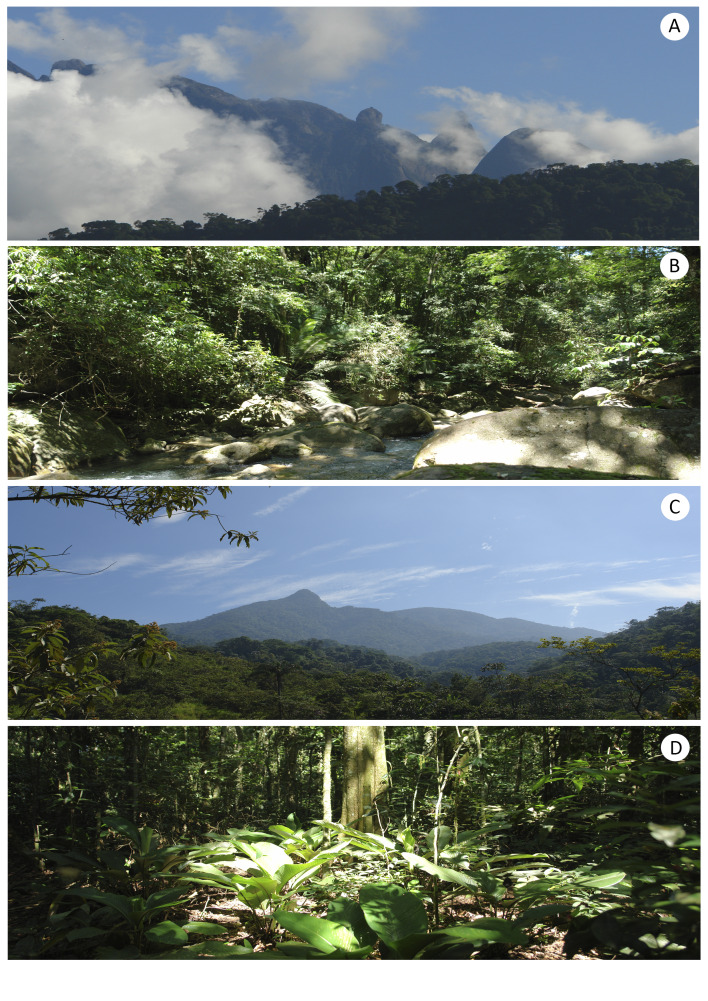
Landscapes of the Reserva Biológica do Tinguá (Rebio Tinguá), Rio de Janeiro, Brazil. **A** View of the 'Pico do Tinguá' peak, **B** Overview of the 'Rio d’Ouro' river within the protected area, **C** Dense Ombrophilous Forest of the Rebio, **D** Overview of the understory within the protected area (Photos: Claudio N. Fraga).

The Rebio Tinguá is a protected area spanning four municipalities of Rio de Janeiro state: Nova Iguaçu, Duque de Caxias, Petrópolis, and Miguel Pereira. Located at the boundary between the Serra do Mar and the Baixada Fluminense region (a lowland area within the greater Rio de Janeiro metropolitan area, encompassing several municipalities—among them Duque de Caxias, Nova Iguaçu, Mesquita, and Belford Roxo—and stands out as a key urban and economic center). Its geographical limits coordinates are 22°22'20" to 22°45'00" S and 43°05'40" to 43°40'00" W (Fig. 2). The Reserve serves as an important watershed divide for the Baía de Guanabara, Baía de Sepetiba, and Rio Paraíba do Sul basins. Covering 26,260 hectares, Rebio Tinguá is the largest Biological Reserve in the state of Rio de Janeiro and the third largest in the southeastern region of Brazil (ICMBio 2024). Its highest point Pico do Tinguá reaches an altitude of 1,600 meters above sea level. The reserve’s history is closely tied to the Baixada Fluminense region’s expansion and Rio de Janeiro city. Established in 1989 (Federal Decree No. 97,780, May 23, 1989) and designated as a Biosphere Reserve by UNESCO in March 1991, Rebio Tinguá is part of the Serra do Mar Biodiversity Corridor and the Central Fluminense Atlantic Forest Mosaic. Alongside other nearby protected areas, its primary goal is to safeguard a portion of the Atlantic Forest and essential natural resources, including watershed areas (ICMBio 2024). The reserve's vegetation consists of Dense Ombrophilous forests ranging from lowland to submontane, montane, and highland vegetation types (IBGE 2012;Fig. 3).

### Coordinates

 and Latitude; and Longitude.

## Taxonomic coverage

### Description

**Figure 4.**
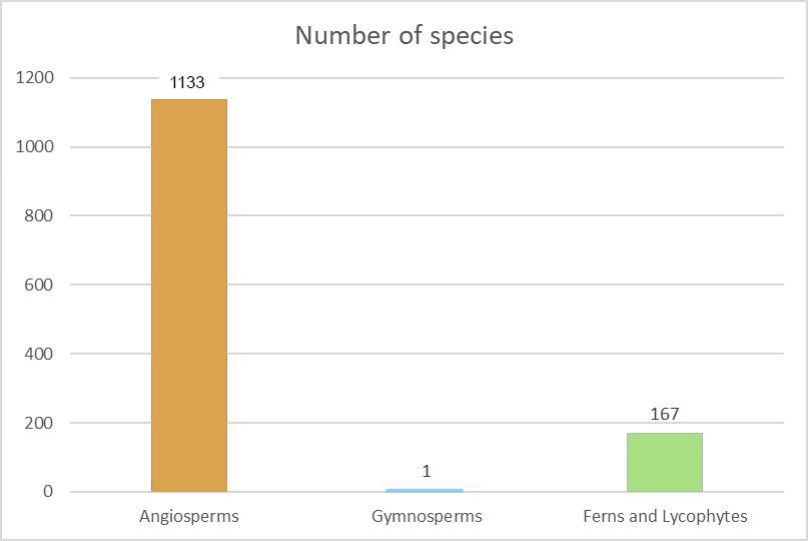
Number of species by plant groups at Reserva Biológica do Tinguá, Rio de Janeiro, Brazil.

The vascular plant list of the Rebio Tinguá includes a total of 1,301 species (see Suppl. material 1) grouped in 572 genera and 147 families. Among these 1,133 are angiosperms (495 genera and 122 families;Fig. 4), one is a gymnosperm *Podocarpussellowii* Klotzsch ex Endl., and 167 are ferns and lycophytes (76 genera and 24 families;Fig. 4). Of these species, 52,2% are endemic to the Atlantic Forest.

**Figure 5.**
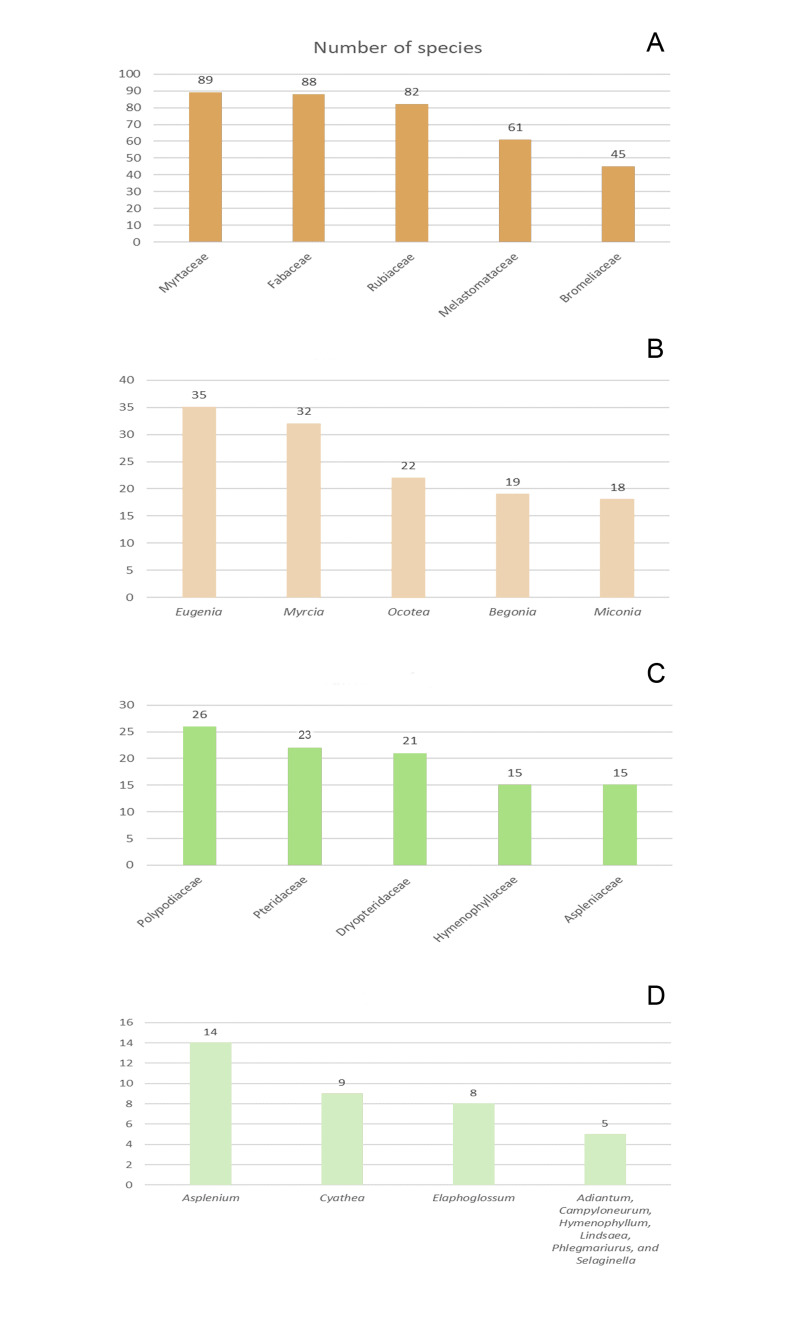
Number of species by plant groups of the Reserva Biológica do Tinguá (Rebio Tinguá), Rio de Janeiro, Brazil: **A.** Richest families of angiosperms, **B.** Richest genera of angiosperms, **C.** Richest families of ferns and lycophytes, and **D.** Richest genera of ferns and lycophytes.

**Figure 6.**
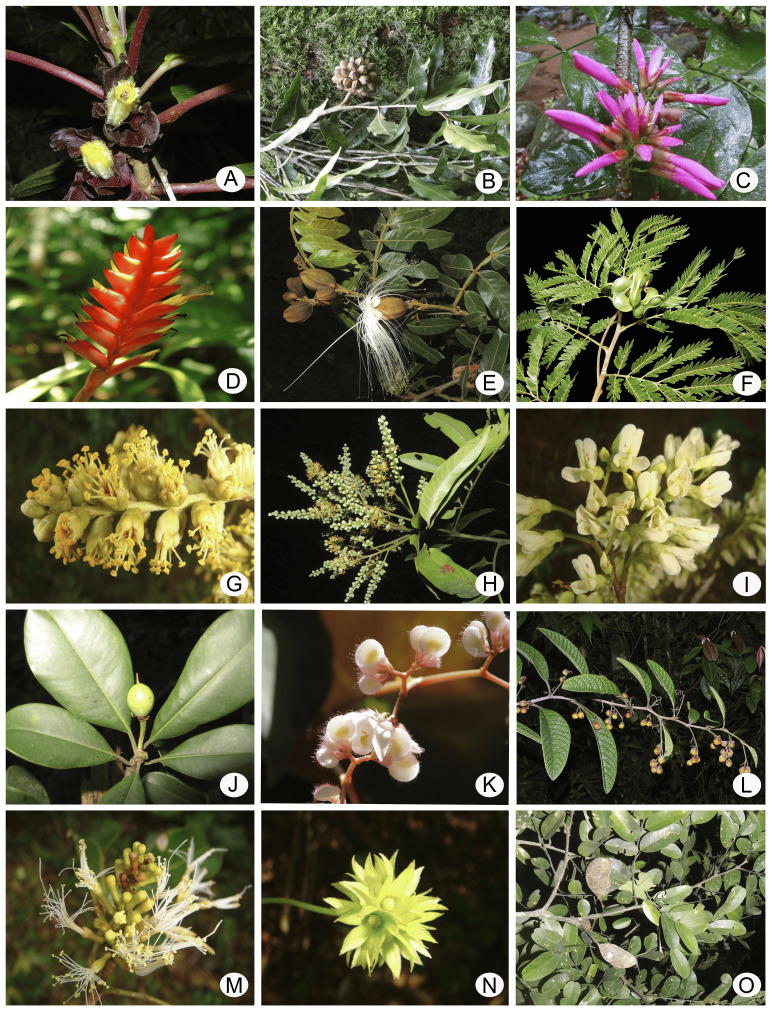
Examples of angiosperms of Reserva Biológica do Tinguá (Rebio Tinguá), Rio de Janeiro, Brazil. **A** *Nematanthushirtellus* (Schott) Wiehler (Gesneriaceae), **B** *Duguetiamicrophylla* (R.E.Fr.) R.E.Fr. (Annonaceae), **C** *Dahlstedtiapinnata* (Benth.) Malme (Fabaceae), **D** *Vrieseagradata* (Baker) Mez (Bromeliaceae), **E** *Ingasessilis* (Vell.) Mart. (Fabaceae), **F** *Jupunbalangsdorffii* (Benth.) M.V.B.Soares, M.P.Morim & Iganci (Fabaceae), **G** *Tachigalibeaurepairei* (Harms) LF.Gomes da Silva & H.C.Lima (Fabaceae), **H** *Myrsinehermogenesii* (Jung-Mend. & Bernacci) M.F.Freitas & Kin.-Gouv. (Primulaceae), **I** *Dalbergianigra* (Vell.) Allemão ex Benth. (Fabaceae), **J** *Manilkarasubsericea* (Mart.) Dubard (Sapotaceae), **K** *Begoniafimbritepala* E.L. Jacques (Begoniaceae), **L** *Davillaglaziovii* Eichler (Dilleniaceae), **M** *Ingalenticellata* Benth. (Fabaceae), **N** *Licaniakunthiana* Hook.f. (Chrysobalanaceae), **O** *Apuleialeiocarpa* (Vogel) J.F.Macbr. (Fabaceae). (Photos: A, L by M.D.F. Araújo; B, H, J, N by P. Rodrigues; C, E, G, I, M by H.C. Lima; D, K by C.N. Fraga; F, O by L.S.J. Deccache).

**Figure 7.**
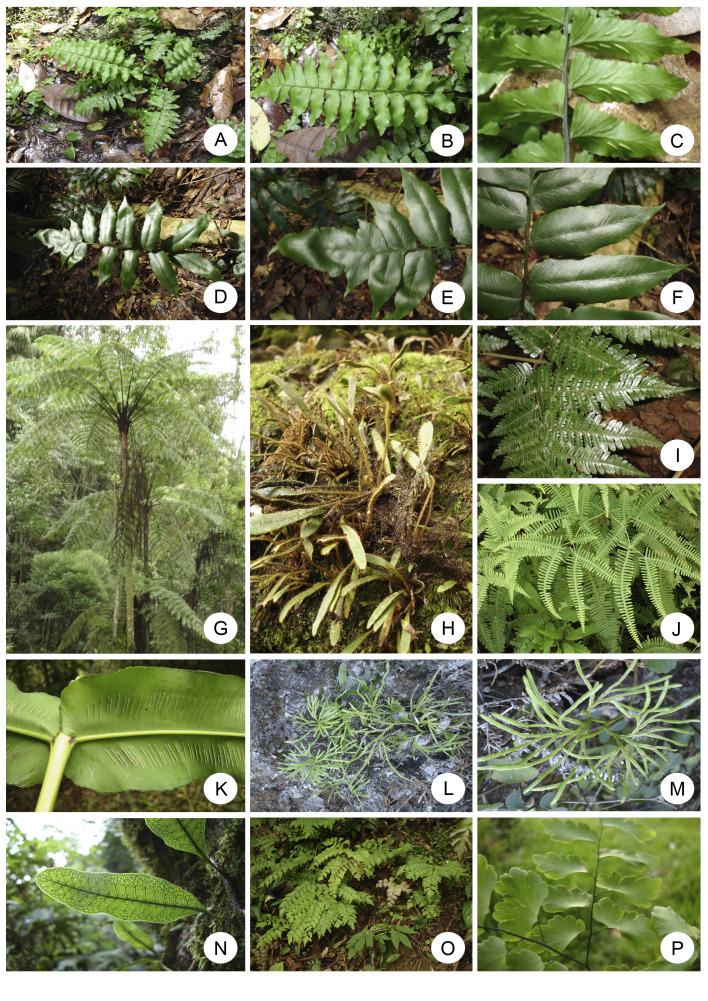
Ferns and lycophytes of Reserva Biológica do Tinguá, Rio de Janeiro (Rebio Tinguá), Brazil. **A, B, C** *Hymenaspleniumtriquetrum* (N. Murak. & R.C. Moran) L. Regalado & Prada (Aspleniaceae), **D, E, F** *Diplaziumfimbriatum* Mynssen & F.B. Matos, **G** *Cyatheadelgadii* Sternb. (Cyatheaceae), **H** *Elaphoglossumhorridulum* (Kaulf.) J.Sm. (Dryopteridaceae), **I** *Megalastruminaequale* (Kaulf. ex Link) A.R.Sm. & R.C.Moran, **J** *Dicranopterisflexuosa* (Schrad.) Underw. (Gleicheniaceae), **K** *Hemidictyummarginatum* (L.) C.Presl (Hemidictyaceae), **L, M** *Diphasiastrumthyoides* (Willd.) Holub (Lycopodiaceae), **N** *Microgrammasquamulosa* (Kaulf.) de la Sota, **O** *Adiantumpentadactylon* Langsd. & Fisch. (Polypodiaceae), **P** *Adiantumpentadactylon* Langsd. & Fisch. (Pteridaceae). (Photos: L.S. Sylvestre).

The richest angiosperm families in Rebio Tinguá are Myrtaceae (89 species), Fabaceae (88), Rubiaceae (82), Orchidaceae (82), Melastomataceae (61) and, Bromeliaceae (45) (Fig. 5a, Fig. 6). Together, these families represent 34.2% (447 species) of the total species found in the Rebio Tinguá. The most species-rich angiosperm genera include *Eugenia* (35 species) *Myrcia* (32), *Ocotea* (22), *Begonia* (19), and *Miconia* (19) (Fig. 5b), representing 9,7% of the total species. These families and genera are also among the ten richest in both Brazil and the Atlantic Forest (BFG 2021). Regarding ferns and lycophytes, the richest families are Polypodiaceae (26 species), Pteridaceae (23), Dryopteridaceae (21), Hymenophyllaceae (15), and Aspleniaceae (15), representing 7,6% of the total species (Fig. 5c, Fig. 7). The richest genera in ferns and lycophytes are *Asplenium* (14), *Cyathea* (9), *Elaphoglossum* (8), *Adiantum*, *Campyloneurum*, *Hymenophyllum*, *Lindsaea*, *Phlegmariurus*, and *Selaginella* (five species each;Fig. 5d). These families are consistently ranked among the richest in Rio de Janeiro state and Brazil (Flora e Funga do Brasil 2025).

Considering the size of the reserve and the number of records, the species richness in this protected area is notably high, even by Atlantic Forest standards. Rebio Tinguá harbors 1,301 species across 26,260 hectares. This is particularly significant when compared to other studies of protected areas in the state of Rio de Janeiro, such as Parque Nacional do Itatiaia (2,316 species in 30,000 hectares - also including bryophyte species;Moreira 2020), Parque Estadual da Pedra Selada (303 species in 8,036 hectares;Waga et al. 2024), and Parque Estadual da Serra da Concórdia (231 species in 5,952 hectares;Deccache et al. 2024b).

The Rebio Tinguá vegetation exhibits a high degree of conservation, with areas of primary forest and secondary vegetation at various stages of regeneration—ranging from initial to intermediate and advanced (MMA–IBAMA 2006). However, some areas have suffered fragmentation and loss of primary vegetation due to anthropogenic activities, which are incompatible with conservation efforts. This is especially evident in the northwestern portion of the protected area, which was historically used as grazing land until recently, underscoring the pressing need for conservation efforts (Deccache 2023).

## Traits coverage

*Origin, endemism and conservation status*

The vascular plant list of Rebio Tinguá comprises 1,285 native species, 12 non-natives, and four cultivated in Brazil. We found 677 endemic species of the Atlantic Forest, of which 582 are angiosperms and 94 are ferns and lycophytes (see Suppl. material 1). The families with the highest number of endemic species to the Atlantic Forest are Myrtaceae (66 species), Orchidaceae (47), Rubiaceae (45), Fabaceae (37), Bromeliaceae, and Melastomataceae (36 each), followed by Polypodiaceae (15), and Dryopteridaceae (14).

The reserve harbors 566 species that have been assessed for their conservation status in Brazil. Among them, 97 species are under threat, five are Critically Endangered (CR), 57 Endangered (EN), and 36 Vulnerable (VU). Notably, 86 species are endemic to the Atlantic Forest (Table 1). Additionally, the reserve hosts 32 species assessed as Near Threatened (NT), 420 as Least Concern (LC), 16 species as Data Deficient (DD), and 735 species as Not Evaluated (NE).

## Temporal coverage

**Single date:** .

### Notes

The botanical collections by A. C. Brade and A. F. M. Glaziou, dating back to the 19^th^ and 20^th^ centuries and housed at the RB and P herbaria, are particularly noteworthy. Similarly, the collections by H. C. de Lima, L. S. Sylvestre, S. J. Silva Neto, and M. G. Bovini, also stored in the RB herbarium, deserve special mention. These collections, a result of collaborative efforts, have significantly expanded our understanding of the Rebio Tinguá flora, demonstrating the power of collective scientific endeavor.

To date, 15 new species from different botanical families have been described based on material collected in the Reserva Biológica do Tinguá. These species are: *Aphelandracrenatifolia* Rizzini (Acanthaceae), *Monsanimatinguaensis* R.Santos & Fontella (Apocynaceae), *Begoniafimbritepala* E.L.Jacques (Begoniaceae), *Jupunbavillosa* (Iganci & M.P.Morim) M.V.B.Soares et al. (Fabaceae), Swartziamyrtifoliavar.elegans (Schott) R.S.Cowan (Fabaceae), *Tachigaliurbaniana* (Harms) L.G.Silva & H.C.Lima (Fabaceae), *Quararibeasimilis* C.D.M. Ferreira & Bovini (Malvaceae), *Leandraquinquedentata* (DC.) Cogn. (Melastomataceae), *Glaziophytonmirabile* Franch. (Poaceae), *Euplassaglaziovii* (Mez) Steyerm. (Proteaceae), *Palicoureaoctocuspis* (Müll. Arg.) C.M. Taylor (Rubiaceae), *Simirawalteri* Silva Neto & Callado (Rubiaceae), *Fagararetusa* Albuq. (Rutaceae), *Solanumverticillatum* S. Knapp & Stehmann (Solanaceae), and *Daphnopsiscoriacea* Taub. (Thymelaeaceae). These findings highlight the Rebio Tinguá as a crucial hotspot for plant diversity and endemism in Brazil.

## Usage licence

### Usage licence

Creative Commons Public Domain Waiver (CC-Zero)

## Data resources

### Data package title

Vascular plant list of Reserva Biológica do Tinguá, Brazil

### Number of data sets

1

### Data set 1.

#### Data set name

Database_Rebio_Tingua_Revised_2

#### Data format

CSV

#### Description

The database contains a list of 1,301 vascular plant species in the Rebio Tinguá, including information on taxonomic names, herbarium vouchers, the source database, the collector's name and number, origin, conservation status, and endemism in the Brazilian Atlantic Forest.

**Data set 1.** 

Column label	Column description
ProtectedArea	Name of the Brazilian Protected Area
PlantGroup	Plant Group (Angiosperms or Ferns and Lycophytes)
Family	Plant family
Genus	Plant genus
Species	Epithet of the species
Author	Name of the species author
TaxonID	Family plant , species name and author
Barcode	Herbarium voucher
Herbarium	Acronym for each herbarium
Database	Source database
CollectorName	Collector's name
CollectorNumber	Sequential number assigned to a specific collection by a botanist or collecting team
Origin	native, cultivated or non-native in Brazil
ConservationStatus	Conservation status according to IUCN and CNCFlora
EndemismAF	Species Endemic or not of Atlantic Forest

## Additional information

**Conclusions and prospects**

The Reserva Biológica do Tinguá is one of the few remaining forested areas in Rio de Janeiro and one of the most important remaining areas of the Atlantic Forest in the region, currently protecting about 97 threatened species, of which 86 are endemic to the domain. Despite this, it has been threatened by its proximity to large urban centers and illegal exploitation activities within the protected area.

Urgent conservation measures and political support are needed for its effective protection. Also, future expeditions are necessary in the Rebio Tinguá to fill the knowledge gaps in the unexplored areas, such as Pico do Tinguá, and will be valuable opportunities to enhance our understanding of the region’s floristic diversity. Access difficulties have restricted botanical surveys due to the steep slopes and watercourses that run from the extreme north to the south of the reserve. Exploring these remote areas in future expeditions could lead to the discovery of more species, further increasing the overall richness of this protected area. The vascular plant inventory of Rebio Tinguá underscores the importance of continuous assessments in Brazil’s protected areas, improving our understanding of biodiversity gaps regarding Brazilian flora and supporting the development of effective conservation strategies.

## Supplementary Material

88F5FC0F-49DA-5F90-B6B0-59CDD2BCF600 10.3897/BDJ.13.e157961.suppl1 Supplementary material 1 Vascular plant list of Reserva Biológica do Tinguá, Brazil Data type Taxonomic names, herbarium vouchers, database, collector's name and number, origin, conservation status, and Atlantic Forest endemism. Brief description The database contains a list of 1,301 vascular plant species in the Rebio Tinguá, including information on taxonomic names, herbarium vouchers, database, collector's name and number, origin, conservation status, and endemism in the Brazilian Atlantic Forest. File: oo_1354641.csv https://binary.pensoft.net/file/1354641 Bochorny T, Quinet A, Castello ACD, Alves-Araújo A, Costa AF, Peixoto AL, Sakuragui CM, Fraga CN, Dalastra CH, Zavatin DA, Gonzaga DR, Ramos E, Jacques EL, Guimarães EF, Lírio EJ, Marcusso GM, Sommer GV, Queiroz GA, Lima HC, Azevedo IHF, Baumgratz JF, Sylvestre LS, Deccache LSJ, Giacomin LL, Pederneiras LC, Marinho LC, Souza MC, Nadruz MNC, Morim MP, Bovini MG, Kaehler M, Barros N, Marques OL, Viana PL, Vinicius-Silva R, Neto SJS, Carrijo TT & Forzza RC

## Figures and Tables

**Table 1.** **Table 1**: List of threatened species in Reserva Biológica do Tinguá, Rio de Janeiro State, Brazil, according to CNCFLora/JBRJ database. (CR= Critically Endangered, EN = Endangered, and VU = Vulnerable).

**Family**	**Species**	**Status**
Acanthaceae	*Odontonemadissitiflorum* (Nees) Kuntze	EN
	*Staurogynebrachiata* (Hiern) Leonard	EN
Annonaceae	*Duguetiamicrophylla* (R.E.Fr.) R.E.Fr.	EN
	*Guatterialatifolia* R.E.Fr.	EN
	*Unonopsisriedeliana* R.E.Fr.	EN
	*Xylopiabrasiliensis* Spreng.	VU
Araceae	*Anthuriumaugustinum* K.Koch & Lauche	EN
	*Anthuriumlhotzkyanum* Schott	VU
	*Philodendronnadruzianum* Sakur.	EN
Arecaceae	*Euterpeedulis* Mart.	VU
Begoniaceae	*Begoniadensifolia* Irmsch.	EN
	*Begoniadentatiloba* A.DC.	EN
Bignoniaceae	*Tabebuiacassinoides* (Lam.) DC.	VU
Boraginaceae	*Cordialatiloba* I.M.Johnst.	EN
Bromeliaceae	*Aechmeafasciata* (Lindl.) Baker	VU
	*Neoregeliacoimbrae* E.Pereira	EN
	*Nidulariumfulgens* Lem.	VU
	*Nidulariumutriculosum* Ule	CR
	*Quesnelialateralis* Wawra	VU
	*Wittrockiasuperba* Lindm.	EN
Calophyllaceae	*Kielmeyerainsignis* Saddi	EN
Celastraceae	*Monteverdiacommunis* (Reissek) Biral	VU
	*Tonteleacorcovadensis* Glaz. ex A.C.Sm.	EN
Chrysobalanaceae	*Couepiaparvifolia* Prance	EN
Cyperaceae	*Rhynchosporapilulifera* Bertol.	CR
Dichapetalaceae	*Stephanopodiumestrellense* Baill.	EN
Dilleniaceae	*Davillaglaziovii* Eichler	EN
Elaeocarpaceae	*Sloaneaobtusifolia* (Moric.) Schum.	EN
Ericaceae	*Agaristauleana* (Sleumer) Judd	VU
Fabaceae	*Apuleialeiocarpa* (Vogel) J.F.Macbr.	VU
	*Dalbergianigra* (Vell.) Allemão ex Benth.	VU
	*Dimorphandraexaltata* Schott	EN
	*Ingamendoncaei* Harms	EN
	*Moldenhawerapolysperma* (Vell.) Stellfeld	VU
	*Muellerafilipes* (Benth.) M.J.Silva & A.M.G.Azevedo	VU
	*Tachigalibeaurepairei* (Harms) LF.Gomes da Silva & H.C.Lima	EN
Gesneriaceae	*Besleriamelancholica* (Vell.) C.V.Morton	VU
	*Sinningiahelleri* Nees	CR
	*Sinningialindleyi* Schauer	EN
Lamiaceae	*Salviarivularis* Gardner	VU
Lauraceae	*Mezilaurusnavalium* (Allemão) Taub. ex Mez	EN
	*Ocoteacatharinensis* Mez	VU
	*Ocoteaodorifera* (Vell.) Rohwer	EN
	*Ocoteatabacifolia* (Meisn.) Rohwer	EN
	*Perseameziana* Rasingam & Karthig.	NE
	*Urbanodendronbahiense* (Meisn.) Rohwer	EN
Lecythidaceae	*Carinianalegalis* (Mart.) Kuntze	EN
Lycopodiaceae	*Phlegmariurussellowianus* (Herter) B.Øllg.	VU
Lythraceae	*Lafoensiaglyptocarpa* Koehne	EN
Malpighiaceae	*Heteropterysfragilis* Amorim	EN
Melastomataceae	*Bertolonialeuzeana* (Bonpl.) DC.	EN
	*Huberiacorymbosa* (Cogn.) Bochorny & R.Goldenb.	EN
	*Huberiaedmundoi* (Brade) Bochorny & R.Goldenb.	CR
	*Merianiaglabra* (DC.) Triana	VU
Meliaceae	*Cedrelafissilis* Vell.	VU
	*Cedrelaodorata* L.	VU
Myristicaceae	*Virolabicuhyba* (Schott ex Spreng.) Warb.	EN
Myrtaceae	*Eugeniadisperma* Vell.	EN
	*Eugeniamacahensis* O.Berg	EN
	*Eugeniamacrobracteolata* Mattos	EN
	*Eugeniapruinosa* D.Legrand	EN
	*Eugeniapulcherrima* Kiaersk.	VU
	*Eugeniatenuipedunculata* Kiaersk.	VU
	*Eugeniavattimoana* Mattos	CR
	*Eugeniavillaenovae* Kiaersk.	EN
	*Eugeniaxanthoxyloides* Cambess.	VU
	*Myrciacarioca* A.R.Lourenço & E.Lucas	VU
	*Myrciafusiformis* (M.L.Kawas.) A.R.Lourenço & E.Lucas	VU
	*Neomitranthesamblymitra* (Burret) Mattos	EN
	*Pliniaedulis* (Vell.) Sobral	VU
Ochnaceae	*Luxemburgiaglazioviana* (Engl.) Beauverd	VU
Orchidaceae	*Epidendrumaddae* Pabst	VU
	*Grandiphyllumdivaricatum* (Lindl.) Docha Neto	VU
	*Pabstiellalingua* (Lindl.) Luer	EN
Passifloraceae	*Passifloraimbeana* Sacco	EN
Poaceae	*Diandrolyratatianae* Soderstr. & Zuloaga	EN
	*Glaziophytonmirabile* Franch.	EN
	*Merostachysburmanii* Send.	EN
Proteaceae	*Roupalagracilis* Meisn.	EN
Pteridaceae	*Doryopterisrediviva* Fée	VU
	*Jamesoniainsignis* (Mett.) Christenh.	EN
Rubiaceae	*Chomeliaestrellana* Müll.Arg.	EN
	*Coussareaaccedens* Müll.Arg.	VU
	*Farameafilamentosa* Müll.Arg.	EN
	*Farameatinguana* Müll.Arg.	CR
	*Psychotriaclavipes* Müll.Arg.	EN
	*Psychotriaglaziovii* Müll.Arg.	VU
	*Psychotriasubspathacea* Müll.Arg.	VU
	*Rudgeaerythrocarpa* Müll.Arg.	EN
	*Rudgeajasminoides* (Cham.) Müll.Arg.	VU
	*Rudgeavellerea* Müll.Arg.	VU
	*Rustiaangustifolia* K.Schum.	EN
	*Rustiagracilis* K.Schum.	EN
	*Simirawalteri* Silva Neto & Callado	EN
Rutaceae	*Zanthoxylumretusum* (Albuq.) P.G.Waterman	EN
Sapindaceae	*Allophylusheterophyllus* (Cambess.) Radlk.	VU
Sapotaceae	*Pouteriabapeba* T.D.Penn.	EN
	*Pouteriacoelomatica* Rizzini	EN
	*Pradosiakuhlmannii* Toledo	EN
Thelypteridaceae	*Goniopterisrefracta* (Fischer & C. Meyer) Brade	EN
